# Impact of preeclampsia/eclampsia on hemorrhagic and ischemic stroke risk: A 17 years follow-up nationwide cohort study

**DOI:** 10.1371/journal.pone.0276206

**Published:** 2022-11-09

**Authors:** Chi-Jou Chuang, Wen-Yen Chiou, Hsuan-Ju Yang, Hon-Yi Lin, Shih-Kai Hung, Moon-Sing Lee, Chia-Hui Chew, Ben-Hui Yu, Feng-Chun Hsu, Liang-Cheng Chen

**Affiliations:** 1 Department of Obstetrics and Gynecology, Dalin Tzu Chi Hospital, Buddhist Tzu Chi Medical Foundation, Chiayi, Taiwan; 2 School of Medicine, Tzu Chi University, Hualien, Taiwan; 3 Department of Radiation Oncology, Dalin Tzu Chi Hospital, Buddhist Tzu Chi Medical Foundation, Chiayi, Taiwan; Tulane University School of Public Health and Tropical Medicine, UNITED STATES

## Abstract

**Background and purposes:**

The long-term risk of stroke in women with preeclampsia/eclampsia is a concerning issue. In this study we further investigated different stroke subtypes and differentiated follow-up time intervals.

**Methods:**

Between 2000 and 2017, 1,384,427 pregnant women were registered in the National Health Insurance Research Database in Taiwan. After excluding women with previous stroke history and exact matching with all confounders, 6,053 women with preeclampsia/eclampsia and 24,212 controls were included in the analysis sample.

**Results:**

Over the 17-year follow-up, the adjusted hazard ratio (aHR) for stroke in women with preeclampsia/eclampsia was 2.05 (95% confidence interval, CI = 1.67–2.52, p<0.001). The 17 years overall aHR of both ischemic and hemorrhagic stroke were 1.98 and 3.45, respectively (p<0.001). The stroke subtypes, hemorrhagic and ischemic, had different time trend risks, and hemorrhagic stroke risks kept higher than that of ischemic stroke. The aHR of ischemic stroke reached a peak during 1–3 years after childbirth (aHR = 3.09). The aHR of hemorrhagic stroke reached a peak during 3–5 years (aHR = 7.49).

**Conclusions:**

Stroke risk persisted even after decades, for both ischemic and hemorrhagic subtypes. Women with preeclampsia/eclampsia history should be aware of the long-term risk of stroke.

## Introduction

Preeclampsia/eclampsia, a hypertensive disorder of pregnancy, is a serious and common obstetric complication [1]. The incidence of preeclampsia/eclampsia in pregnant women is 2–8% worldwide, and it is a major cause of maternal morbidity and mortality [2–4]. Severe preeclampsia/eclampsia is a serious threat to the lives of mothers and fetuses, leading to cardiovascular and metabolic complications for mothers and persistent bradycardia, hypotension, and neonatal hypoglycemia for fetuses [5–7]. Preeclampsia/eclampsia may also lead to serious kidney, brain, and blood clotting problems [8, 9]. Overall, 10%-15% of direct maternal deaths are associated with preeclampsia and eclampsia in low- and middle-income countries [4].

Stroke is an important issue not only in the elderly, but also in pregnant women. It has been reported that the incidence of stroke, both ischemic and hemorrhagic subtypes, is increased in the peripartum and postpartum stages [10–15]. The incidence of stroke ranges from 1.5 to 34.2 per 100,000 deliveries in pregnancy and the puerperium [16, 17]. It has been reported that preeclampsia/eclampsia-related stroke can cause direct obstetric death [18, 19].

The incidence of stroke in pregnant women in previous studies is varied due to different preexisting risk factors [16, 20]. However, the long-term stroke risk in term of different time intervals after childbirth is not well studied. In this study, we aimed to utilize a nationwide database to explore the long-term stroke risk, differentiating follow-up time intervals for both of ischemic and hemorrhagic stroke subtypes, in women with a history of preeclampsia/eclampsia after 17 years follow-up.

## Materials and methods

This study was conducted following the Declaration of Helsinki. The study protocol was also approved by the Institutional Review Board (IRB) of Dalin Tzu Chi Hospital of Buddhist Tzu Chi Medical Foundation (approval number, B10402022). The IRB absolved the study from the requirement for written informed consent due to no direct contact with individual patients from this de-identified database.

### Data source and availability

We used the Taiwan National Health Insurance Research Database (NHIRD) to analyze the incidence rate of stroke in women with preeclampsia/eclampsia and compared it to those without preeclampsia/eclampsia. The information contained within the database was released for research purposes by the Health and Welfare Data Science Center (HWDC), Ministry of Health and Welfare, Taiwan. The raw data from the NHIRD is available to the research community; however, the data must be analyzed within the HWDC after the study proposal is approved (https://dep.mohw.gov.tw/dos/np-2497-113.html). The confidentiality assurances were addressed by following the data regulations of the HWDC. The study protocol, analytic methods, and statistical programming codes are available from the corresponding author on reasonable request.

Taiwan NHIRD contains all the records of diagnosis and treatment of approximately 99% of people from inpatient, outpatient, and emergency departments [21]. The data collection of pregnant women from the Taiwan NHIRD ranged from 2000 to 2017, and it was included in this study for statistical analysis. The data included were evaluated by the National Health Insurance Administration (NHIA) quarterly expert reviews on every 50 to 100 ambulatory and inpatient claims filed by each medical institution [22]. False diagnostic reports are subject to severe penalties from the NHIA [23].

Records of pregnant women in this database were collected and categorized into two groups of women, those with and without preeclampsia/eclampsia. Based on the International Classification of Disease, Ninth Revision, Clinical Modification (ICD-9-CM) codes for pregnant women, the codes were 650, 651, 652, and 653, while those for pregnant women with preeclampsia/eclampsia were 642.4, 642.5, 642.6, and 642.7.

**Fig 1:**
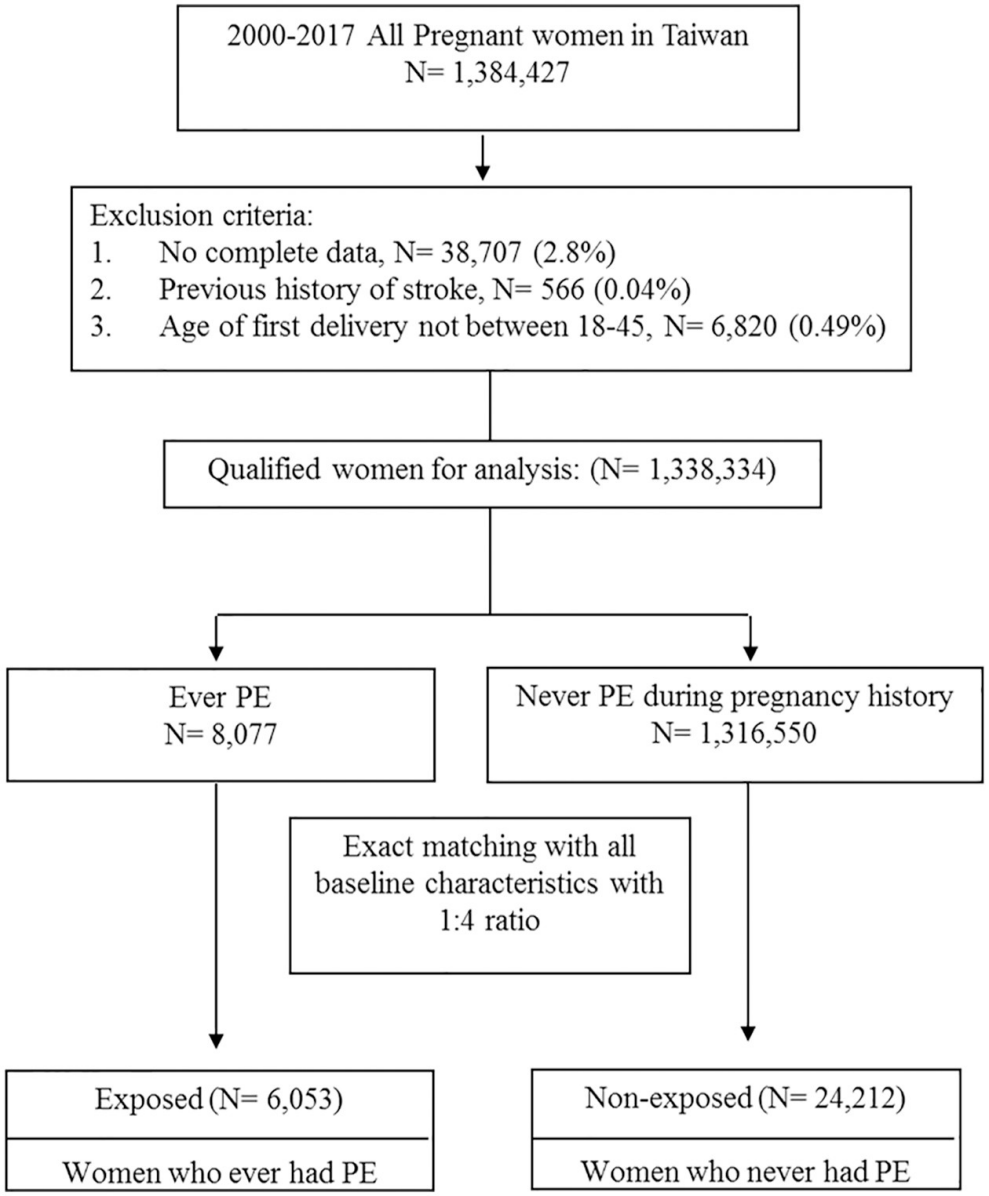
A flow chart illustrating the selection procedure of study subjects. PE; preeclampsia/eclampsia.

Between 2001 and 2017, 1,384,427 pregnant women with delivery were registered in the Taiwan NHIRD. Fig 1 shows our study’s flow diagram. We excluded 38,707 cases with missing confounders and 566 cases with stroke history. In addition, we only included women with the delivery age between 18 to 45 years. Finally, we enrolled 1,338,334 cases in this study, divided into groups of pregnant women with (N = 8,077) and without (N = 1,316,550) preeclampsia/eclampsia.

Because of the large number of women in our nationwide database, we performed exact matching for every subject and control subject. Every woman with preeclampsia/eclampsia had 4 matched control subjects. Every woman and her corresponding matched control subjects had the same age, delivery type, gestation number, hospital level, delivery season, living area, family income level and all comorbidities were the same. Comorbidities included chronic HTN, gestational diabetes, anemia, antepartum hemorrhage, and postpartum hemorrhage. Therefore, the chi-square test for these two matched cohorts reveal all baseline characteristics (S1 Table) had p value of 1.000. After 1:4 exact matching, 6,053 cases were selected in the preeclampsia/eclampsia group and 24,212 in the non-preeclampsia/eclampsia group.

The primary outcomes included: codes for hemorrhagic stroke being 430, 431, 432, and codes for ischemic stroke being 433, 434, 435, 436, and 437. The potential confounders considered in this study were age, type of delivery (cesarean section or normal spontaneous delivery), multiple gestation, hospital level, season during delivery, comorbidities, and sociodemographic variables (Table 1 and S1 Table). To more accurately evaluate the effect of preeclampsia/eclampsia on stroke, the above matched confounders were incorporated into multivariate analysis for adjustment, because they may still affect the stroke risk estimation. For example, hypertension and old age, etc, would still increase the risk of stroke, even these two cohort groups had same distribution of hypertension and age. If these potential confounders are not adjusted for, the estimated stroke hazard ratio of preeclampsia/eclampsia might be overestimated.

**Table 1:** Adjusted hazard ratios of stroke in women with preeclampsia in Taiwan, 2000–2017.

aHR for stroke	aHR	95% CI
Preeclampsia	2.05***	1.67–2.52
Age at delivery (<30, ref.)		
30–32	1.37*	1.02–1.83
32–35	1.37*	1.01–1.85
>35	2.61***	2.03–3.36
Caesarean section	1.08	0.89–1.31
Multiple gestation	1.00	0.71–1.41
Hospital level (Medical center, ref.)		
Regional hospital	1.19	0.89–1.59
Local hospital	1.11	0.83–1.49
Clinics	1.02	0.75–1.38
Season of delivery		
Summer	1.24	0.94–1.62
Autumn	1.14	0.87–1.51
Winter	1.07	0.81–1.40
Comorbidities		
Hypertension	3.35***	1.99–5.63
Gestational diabetes mellitus	0.88	0.33–2.38
Anemia	1.24	0.75–2.05
Antepartum hemorrhage	2.10*	1.10–4.01
Postpartum hemorrhage	1.13	0.15–8.55
Geographic region (North, ref.)		
Central	0.98	0.75–1.27
East	1.42	0.79–2.55
South	0.94	0.74–1.21
Urbanization level (Metropolis, ref.)		
Satellite cities	1.03	0.80–1.33
Rural areas	1.23	0.91–1.66
Family income (low, ref.)		
Median	0.62***	0.47–0.82
High	0.63**	0.47–0.84
Highest	0.61**	0.45–0.84

aHR, adjusted hazard ratio; 95% CI, 95% confidence interval;

*p< 0.05;

** p< 0.01,

*** p< 0.001;

ref., reference; The adjusted hazard ratios in this Table 1 were from the multivariate Cox model that included all of the variables listed.

The hospital level was included in the analysis, account for different care qualities during pregnancy at different hospital levels. Socioeconomic variables, including geographic region, urbanization level, and monthly income-based insurance premiums were analyzed to reduce bias resulting from lifestyle. The follow-up time in this study was from 2000 to 2017. The incidence of stroke compared in these two cohorts was listed in Table 2.

**Table 2:** Stroke incidence rates in women with and without preeclampsia history in Taiwan, 2000–2017.

Variables	Women with and without preeclampsia after 1:4 matching		P value
	Preeclampsia N = 6,053 (%)	Non- preeclampsia N = 24,212 (%)	
Stroke*	130 (2.15)	298 (1.23)	<0.001
Ischemic stroke	114 (1.88)	271 (1.12)	<0.001
Hemorrhagic stroke	33 (0.55)	44 (0.18)	<0.001

*Some patients had experienced both ischemic and hemorrhagic strokes at different times.

Because our database has a very long time span of 17 years, we further divided the overall follow up time into several sub-intervals of follow up period. Reviewing the literature, previous studies reported the stroke risk for 3 months antepartum, and 3 days, 6 weeks, 6 months, and 12 months postpartum [14]. For follow up time exceeded one year after delivery, only one longitudinal cohort study recently had ever reported the later-life stroke incidence [24], but their study did not compare stroke risk in different time intervals. Because no specific time division criteria could be referred, we conventionally divided the follow-up time into 0–5, 5–10, and 10–15 years, and further divided the 0–5 years close to delivery into 0–1, 1–3, and 3–5 years.

To further evaluate risks in these different sub-intervals, we computed conditional probabilities, conditioning on not having a stroke by the beginning of this sub-interval of the follow-up period; that is, women who had a stroke episode by the beginning of the specific sub-interval were excluded. Then we re-did exact matching for these new cohorts in each sub-interval, and performed several corresponding multivariate Cox models for each sub-interval (Table 3). Table 3 shows the aHRs which were computed from new re-matched cohorts for these sub-intervals and several multivariate Cox models to evaluate the stroke risks that might occur during these sub-intervals.

**Table 3:** Stroke risks of women with and without preeclampsia/eclampsia in different sub-intervals of the follow-up period.

Stroke Type		Interval (year)	aHR	95% CI
Stroke	short	0–1	1.65	0.85–3.25
		1–3	3.20***	1.82–5.63
		3–5	2.24**	1.22–4.16
	intermediate	5–10	2.17***	1.53–3.10
	long	10–15	1.80**	1.20–2.70
	overall	0–17	2.05***	1.67–2.52
Ischemic stroke	short	0–1	1.82	0.86–3.85
		1–3	3.09***	1.71–5.58
		3–5	1.95*	1.02–3.72
	intermediate	5–10	2.12***	1.47–3.07
	long	10–15	1.58*	1.02–2.47
	overall	0–17	1.98***	1.59–2.46
Hemorrhagic stroke	short	0–1	2.28	0.66–7.87
		1–3	4.60*	1.17–18.03
		3–5	7.49*	1.18–47.33
	intermediate	5–10	4.93***	2.17–11.22
	long	10–15	3.13**	1.34–7.30
	overall	0–17	3.45***	2.18–5.47

aHR, adjusted hazard ratio; 95% CI, 95% confidence interval;

*p< 0.05;

** p< 0.01,

*** p< 0.001

### Statistical methods

The basic characteristics between the two study groups were compared by Chi-square test (S1 Table). The incidences of stroke in these two cohorts were also compared (Table 2). We used a multivariate Cox regression model, adjusting all the potential confounding factors, to obtain the adjusted hazard ratio (aHR) and 95% confidence interval (CI) for stroke occurrence (Table 1). Cumulative incidence functions for the first occurrence of stroke episode between preeclampsia/eclampsia and non-preeclampsia/eclampsia women were compared using the Kaplan–Meier method. The 17-years follow-up duration was further divided into different sub-intervals. An adjusted hazard ratio was conditionally estimated based on women still being followed and not having a stroke by the beginning of those sub-intervals of the follow-up period, by several multivariate Cox models for each sub-intervals. SAS software (version 9.2; SAS Institute, Inc., Cary, NC) was used for all statistical analyses. A two-sided *P*-value of <0.05 was considered statistically significant.

## Results

The mean ages of the groups with and without preeclampsia/eclampsia were 32.04 and 31.84 years, respectively. No statistically significant differences were found in distribution of the all demographic characteristics and comorbidities between two groups after exact matching.

Regarding the risk for all stroke, preeclampsia/eclampsia women had higher all stroke episodes than non-preeclampsia/eclampsia women (2.15% versus 1.23%, p< 0.001, Table 2). After distinguishing incidences for different stroke subtypes, the incidence rates of both ischemic and hemorrhagic stroke subtypes in the preeclampsia/eclampsia group were higher than that in the non-preeclampsia/eclampsia group (1.88% versus 1.12%, 0.55% versus 0.18%, respectively, both p< 0.001, Table 2).

Table 1 presents the aHRs of stroke after adjusting for covariates. Overall, stroke risk was significantly higher in the preeclampsia/eclampsia group than in the non-preeclampsia/eclampsia group (aHR = 2.05, 95% CI = 1.67–2.52, *p*< 0.001). Women aged >35 years had higher risk of stroke than that of women who aged <30 years (aHR = 2.61, CI = 2.03–3.36, *p*< 0.001). Variables such as cesarean section, multiple gestations, hospital level, and the season of maternal delivery did not have significant risk for stroke. For comorbidities, hypertension increased risks for stroke with aHR 3.35 (95% CI = 1.99–5.63, *p*<0.001) in these two matched cohorts. APH had a significant risk for stroke (aHR = 2.10, CI = 1.10–4.01, *p* = 0.02). Other comorbidities such as GDM, anemia, and PPH had no significant impact on stroke occurrence. Socioeconomic variables, such as geographic region and urbanization level, had no significant stroke risk, except family income. Women in median or high-income families had a lower risk of stroke than that of women in low-income families.

**Fig 2:**
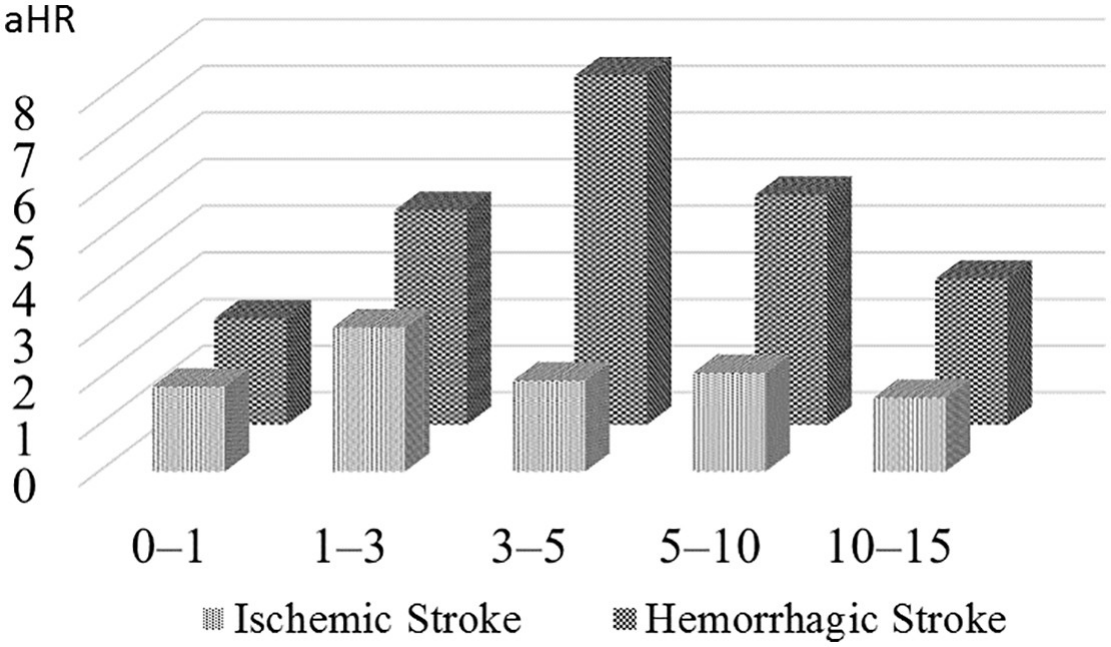
The risk time trends of ischemic and hemorrhagic stroke in women with pre-eclampsia/eclampsia.

Table 3 presents the aHRs of ischemic stroke and hemorrhagic stroke after adjusting for covariates. The aHR of ischemic stroke was 1.98 (95% CI = 1.59–2.46, *p*< 0.001) and the aHR of hemorrhagic stroke was 3.45 (95% CI = 2.18–5.47, *p*< 0.001), respectively. We also observed stroke risk in women with preeclampsia/eclampsia at intervals of 0–1, 1–3, 3–5, 5–10, and 10–15 years (see Table 3). Overall, aHR of stroke reached a peak within 1–3 years after child-birth (aHR 3.20, 95% CI = 1.82–5.63, *p*<0.001). The risk time trends for these two stroke subtypes were different (Table 3 and as illustrated in Fig 2). The aHR of ischemic stroke reached a peak within 1–3 years after childbirth (aHR 3.09, 95% CI = 1.71–5.58, *p*<0.001), while aHR of hemorrhage stroke reached a peak within 3–5 years after childbirth (aHR 7.49, 95% CI = 1.18–47.33, *p* = 0.032). In addition, aHR of hemorrhagic stroke was higher than that of the ischemic stroke at each follow-up time interval.

**Fig 3:**
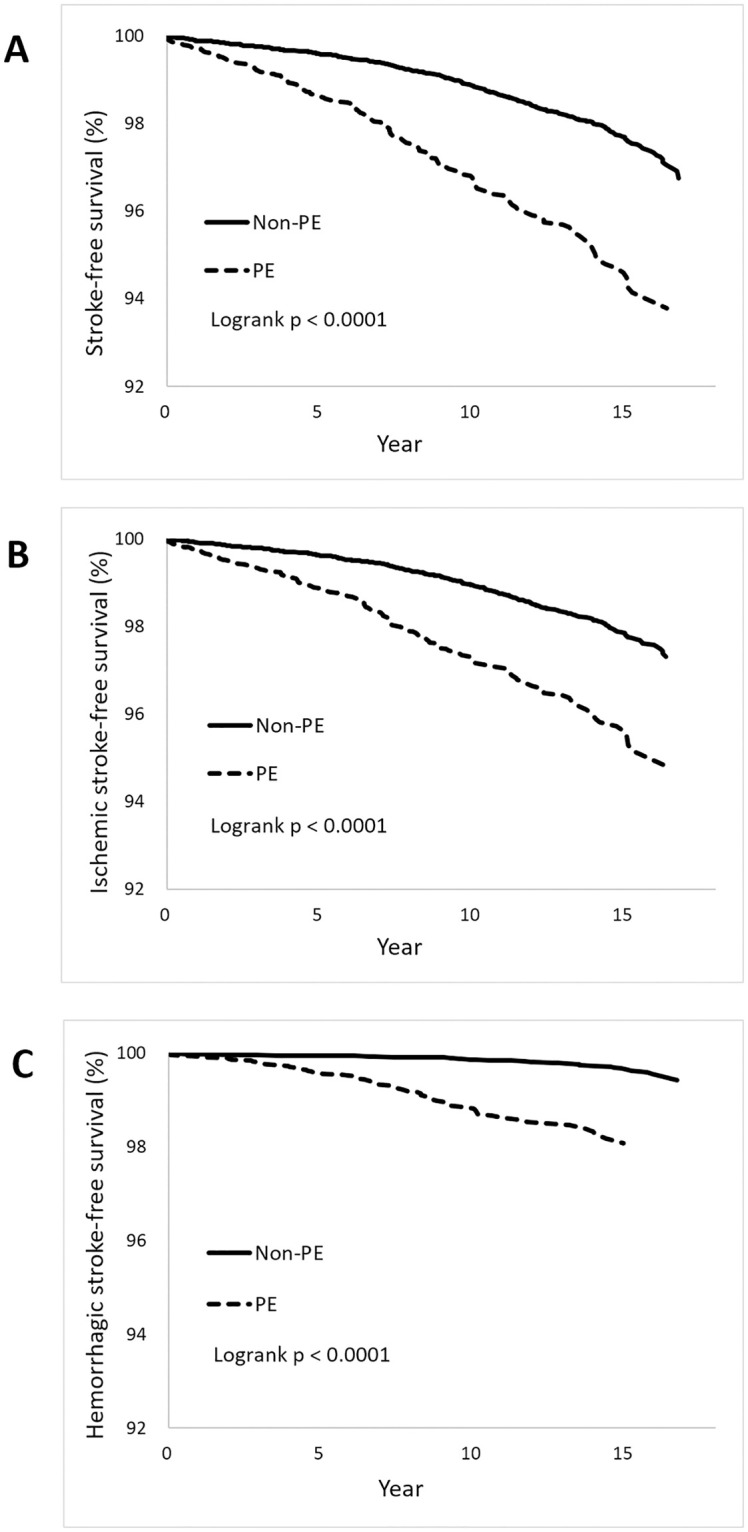
Stroke free survival rate among women with and without preeclampsia/eclampsia for A. overall stroke. B. Ischemic stroke. C. Hemorrhagic stroke. PE; preeclampsia/eclampsia.

The stroke-free-survival rate was significantly higher in women without preeclampsia/eclampsia (97.68%), compared to those with preeclampsia/eclampsia (94.48%). The 15-year ischemic stroke-free and hemorrhagic stroke-free survival rate were all higher in women with preeclampsia/eclampsia than that in women without preeclampsia/eclampsia (97.84%% versus 95.50%, 99.65% versus 98.07%, both *p* < 0.001). Overall, the trend of ischemic stroke-free-survival rate dropped more rapidly and earlier than hemorrhagic free survival rate (Fig 3B and 3C).

## Discussion

Our results show that a history of preeclampsia/eclampsia during pregnancy significantly increased the risk of stroke, both ischemic and hemorrhagic stroke subtypes, in the 17 years after childbirth. In this long-term study, after adjusting for potential confounding variables, women with history of preeclampsia/eclampsia had a 2.05-fold higher stroke risk, 1.98-fold higher ischemic stroke risk and up to 3.45-fold higher hemorrhagic stroke risk than those without. Our findings suggest that long-term stroke risk is a great concern for women with a past episode of preeclampsia/eclampsia.

For pregnancy without preeclampsia/eclampsia, pregnancy itself alone was not a risk factor of stroke during pregnancy, but was a risk of stroke in the postpartum period [25]. In the classic study by Kittner et al., they showed that the adjusted relative risks of ischemic stroke and hemorrhagic stroke, adjusted for age and race, were 8.7 and 28.3 in the six weeks after delivery, but not increased during the pregnancy [25].

Besides the pregnancy process itself, there are several factors which might affect the occurrence and prognosis of stroke, such as hypertension and age. Previous studies reported that population with hypertension had more obvious intracranial occlusion and worse stroke outcome compared to those without hypertension [26–29]. Studies also showed that both of the elevation of systolic and diastolic blood pressure increased the stroke risk for women [30, 31]. Recent study identified genes of predisposing to hypertension associated with preeclampsia/eclampsia in Asian women [32]. In this study, our results confirm the role of hypertensive disease on stroke with a significant aHR of 3.35 (95% CI 1.99–5.63, p< 0.001).

Age is another significant risk factor for stroke. Many obstetric-focused studies discussed the impact of age on maternal stroke risk [13, 33, 34]. For example, women age>39 years was a significant hemorrhagic and ischemic stroke risk factor in a study of postpartum stroke risk [34]. Our results also show that the older women were significantly higher stroke risk they had.

In this study, low family income is another significant risk factor for stroke. Reviewing the literature, previous studies showed women with lower socioeconomic status tended to receive prenatal care less frequently and were at higher risk for obstetric complications [35–37]. Previous studies suggested that socioeconomic disadvantage was associated with increased stroke incidence, severity and mortality at young age population [38, 39].

Many studies evaluated the stroke risk during the pregnancy period [11, 40–45], puerperium [42], and postpartum [40, 46]. Among these risk factors evaluated in the studies, preeclampsia/eclampsia was one of the risk factors which might increase the incidence of stroke. Several studies suggested that preeclampsia/eclampsia would increase stroke risk during the pregnancy period [47, 48], and peripartum [14]. Reviewing the literature, Tang et al. reported the only one study to evaluate the stroke risk in the peripartum period, including pregnancy and postpartum, in women with preeclampsia-eclampsia [14]. The study of Tang et al. followed up pregnant women to the first year post delivery. They evaluated the stroke risk within one year after delivery and found that the adjusted relative risk (aRR) of hemorrhagic stroke were 10.68, 6.45,5.61,11.76 and19.90 for 3 months antepartum, and 3 days, 6 weeks, 6 months, 12 months postpartum, respectively [14]. They also reported the aRR of ischemic stroke were as high as 40.86 within 3 months antepartum, then decreased to 4.35 during 6 months to 12 months postpartum [14].

Compared to Tang’s study, our cohorts study followed women to the 17 years after childbirth. Similarly to that study, our findings show that both ischemic and hemorrhagic stroke risk had a marked ascending trend shortly after childbirth in women with preeclampsia/eclampsia. Besides, our long follow up study shows that the aHR of ischemic and hemorrhagic stroke increased to the peak around within 3 to 5 years after childbirth. The aHR of hemorrhagic stroke reached a peak 3−5 years after childbirth (aHR 7.49), then gradually decreased to around 3-fold. The aHR of ischemic stroke reached a peak 1−3 years after childbirth (aHR 3.09), then gradually decreased to around 1.6-fold. In our study, the ischemic stroke risk reached the peak earlier than hemorrhagic stroke. It is worth noting that, even 10 to 15 years after childbirth, the stroke risk for women with a past history of preeclampsia/eclampsia was still significantly higher than those without preeclampsia/eclampsia (Table 3 and Fig 2).

It has been reported that preeclampsia/eclampsia was independently associated with an increased risk of future heart failure (RR, 4.19), and cardiovascular disease death (RR, 2.21) [12]. During pregnancy, the physiological system of pregnant women is under high stress in order to accommodate the growing fetus. Among women with preeclampsia/eclampsia, previous studies reported the physiological impairments in maternal endothelial dysfunction and changes in vascular tissue structure were found to be associated with stroke risk [11, 49–51]. Left ventricular remodeling/hypertrophy was found to be an adaptive response to minimize wall stress from the development of hypertension during pregnancy in preeclampsia [52]; this altered LV geometry may result in impaired LV function and cardiovascular implications that would persist many years after delivery[53, 54]. This might explain the long term elevated stroke risk in women with preeclampsia/eclampsia.

### Strengths and limitations

First, this study’s major strength is exploring the association between preeclampsia/eclampsia and stroke in a national scope cohort study. Second, this study reported the two different stroke subtypes and explored the corresponding stroke risks in different follow-up sub-intervals for those women who did not have stroke episodes before that specific sub-interval. Finally, because of the nationwide huge database, we used the exact matching method to increase the comparability of those cohorts, and adjusted for potential confounders, to make the stroke risk estimation more accurate.

This study has several limitations. First, this is a retrospective study and treatment of hypertension may have changed during the long follow-up time of the study. This may have an influence on stroke risk, even though we have adjusted the factor of hypertension in the study. Second, this retrospective study did not distinguish preeclampsia from eclampsia. Because our nationwide database is built for health insurance purpose and women with an insurance diagnosis of eclampsia may have or have not preeclampsia diagnosed earlier during pregnancy, separate estimation of stroke risk for women with pre-eclampsia and eclampsia might not be precise. Further clinical study evaluating the stroke risk of pre-eclampsia and eclampsia individually is needed. Third, our health insurance database provides no information on laboratory data, nor the severity of preeclampsia/eclampsia. In addition, smoking habits, alcohol consumption, and body weight/body mass index are not available in our database. Therefore, we could not evaluate the effects of these factors on stroke.

## Conclusions

In summary, our results indicate that the ischemic and hemorrhagic stroke risk of women with preeclampsia/eclampsia history remained high for a long time. Women with preeclampsia/eclampsia history should be aware of stroke risk, even 10 to 15 years after childbirth.

## Supporting information

S1 Table Baseline characteristics of women with and without preeclampsia history in Taiwan, 2000–2017. (DOCX)
