# Supplementary material for: Impact of preeclampsia/eclampsia on hemorrhagic and ischemic stroke risk: A 17 years follow-up nationwide cohort study
Source: PLoS One. 2022 Nov 9;17(11):e0276206. doi: 10.1371/journal.pone.0276206 (PMC9645593; doi:10.1371/journal.pone.0276206)
Supplement: S1 Table — (DOCX) [file pone.0276206.s001.docx]

Table1. Baseline characteristics of women with and without preeclampsia history in Taiwan, 2000-2017.

| Variables | Preeclampsia  N = 6,053 (%) | Non- preeclampsia  N = 24,212 (%) | P value |
| --- | --- | --- | --- |
| Age group of delivery |  |  | 1.000 |
| <30 | 1,913 (31.60) | 7,652 (31.60) |  |
| 30-32 | 1,284 (21.21) | 5,136 (21.21) |  |
| 33-35 | 1,329 (21.96) | 5,316(21.96) |  |
| >35 | 1,527 (25.23) | 6,108 (25.23) |  |
| Caesarean section | 2,967 (49.02) | 11,868 (49.02) | 1.000 |
| Multiple gestation | 601 (9.93) | 2,404 (9.93) | 1.000 |
| Hospital level |  |  | 1.000 |
| Medical center | 1,118 (18.47) | 4,472 (18.47) |  |
| Regional hospital | 1,402 (23.16) | 5,608 (23.16) |  |
| Local hospital | 1,940 (32.05) | 7,760 (32.05) |  |
| Clinics | 1,593 (26.32) | 6,372 (26.32) |  |
| Season of delivery |  |  | 1.000 |
| Spring | 1,395 (23.05) | 5,580 (23.05) |  |
| Summer | 1,491 (24.63) | 5,964 (24.63) |  |
| Autumn | 1,583 (26.15) | 6,332 (26.15) |  |
| Winter | 1,584 (26.17) | 6,336 (26.17) |  |
| Comorbidities |  |  |  |
| Hypertension | 81 (1.34) | 324 (1.34) | 1.000 |
| Gestational diabetes mellitus | 75 (1.24) | 300 (1.24) | 1.000 |
| Anemia | 319 (5.27) | 1,276 (5.27) | 1.000 |
| Antepartum hemorrhage | 86 (1.42) | 344 (1.42) | 1.000 |
| Postpartum hemorrhage | 13 (0.21) | 52 (0.21) | 1.000 |
| Geographic region |  |  | 1.000 |
| North | 2,592 (42.82) | 10,368 (42.82) |  |
| Central | 1,389 (22.95) | 5,556 (22.95) |  |
| South | 1,810 (29.90) | 7,240 (29.90) |  |
| East and remote islands | 96 (1.59) | 384 (1.59) |  |
| Urbanization level |  |  | 1.000 |
| Metropolis | 1,188 (19.63) | 4,752 (19.63) |  |
| Satellite cities | 3,372 (55.71) | 13,488 (55.71) |  |
| Rural areas | 1,327 (21.92) | 5,308 (21.92) |  |
| Family income |  |  | 1.000 |
| Low | 708 (11.70) | 2,832 (11.70) |  |
| Median | 2,255 (37.25) | 9,020 (37.25) |  |
| High | 1,816 (30.00) | 7,264 (30.00) |  |
| Highest | 1,274 (21.05) | 5,096 (21.05) |  |
